# 3,4-Dimethyl-2-(2-oxo-2-phenyl­eth­yl)-2*H*,4*H*-pyrazolo­[4,3-*c*][1,2]benzothia­zine-5,5-dione

**DOI:** 10.1107/S1600536812002188

**Published:** 2012-01-21

**Authors:** Sana Aslam, Hamid Latif Siddiqui, Matloob Ahmad, Iftikhar Hussain Bukhari, Masood Parvez

**Affiliations:** a Institute of Chemistry, University of the Punjab, Lahore 54590, Pakistan; b Chemistry Department, Govt. College University, Faisalabad, Pakistan; c Department of Chemistry, The University of Calgary, 2500 University Drive NW, Calgary, Alberta, Canada T2N 1N4

## Abstract

In the title mol­ecule, C_19_H_17_N_3_O_3_S, the heterocyclic thia­zine ring adopts a half-chair conformation with the S and N atoms displaced by 0.530 (5) and 0.229 (6) Å, respectively, on opposite sides of the mean plane formed by the remaining ring atoms. The ethanone group lies at an angle of 3.8 (3)° with respect to the benzene ring, which lies almost perendicular to the pyrazole ring, with a dihedral between the two planes of 89.22 (11)°. Weak inter­molecular C—H⋯O hydrogen-bonding inter­actions are present.

## Related literature

For the biological activity of pyrazoles, see: Farag *et al.* (2008 ▶); Ciciani *et al.* (2008 ▶); Cunico *et al.* (2006 ▶); Ahmad *et al.* (2010 ▶). For related structures, see: Siddiqui *et al.* (2008 ▶).
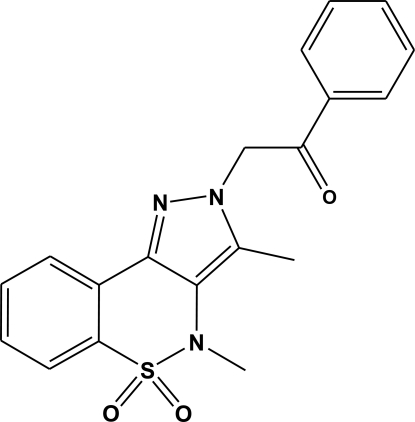


## Experimental

### 

#### Crystal data

C_19_H_17_N_3_O_3_S
*M* *_r_* = 367.42
Monoclinic, 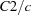

*a* = 24.380 (6) Å
*b* = 11.141 (4) Å
*c* = 14.996 (5) Å
β = 120.76 (2)°
*V* = 3500.1 (19) Å^3^
*Z* = 8
Mo *K*α radiation
μ = 0.21 mm^−1^
*T* = 200 K
0.12 × 0.10 × 0.08 mm

#### Data collection

Nonius KappaCCD diffractometer
Absorption correction: multi-scan (*SORTAV*; Blessing, 1997 ▶) *T*_min_ = 0.975, *T*_max_ = 0.983
12615 measured reflections
3970 independent reflections
2847 reflections with *I* > 2σ(*I*)
*R*_int_ = 0.073

#### Refinement

*R*[*F* ^2^ > 2σ(*F* ^2^)] = 0.069
*wR*(*F* ^2^) = 0.146
*S* = 1.15
3970 reflections
237 parameters
H-atom parameters constrained
Δρ_max_ = 0.27 e Å^−3^
Δρ_min_ = −0.44 e Å^−3^

### 

Data collection: *COLLECT* (Hooft, 1998 ▶); cell refinement: *DENZO* (Otwinowski & Minor, 1997 ▶); data reduction: *SCALEPACK* (Otwinowski & Minor, 1997 ▶); program(s) used to solve structure: *SHELXS97* (Sheldrick, 2008 ▶); program(s) used to refine structure: *SHELXL97* (Sheldrick, 2008 ▶); molecular graphics: *ORTEP-3 for Windows* (Farrugia, 1997 ▶); software used to prepare material for publication: *SHELXL97*.

## Supplementary Material

Crystal structure: contains datablock(s) global, I. DOI: 10.1107/S1600536812002188/pk2383sup1.cif

Structure factors: contains datablock(s) I. DOI: 10.1107/S1600536812002188/pk2383Isup2.hkl

Supplementary material file. DOI: 10.1107/S1600536812002188/pk2383Isup3.cml

Additional supplementary materials:  crystallographic information; 3D view; checkCIF report

## Figures and Tables

**Table 1:** Hydrogen-bond geometry (Å, °)

*D*—H⋯*A*	*D*—H	H⋯*A*	*D*⋯*A*	*D*—H⋯*A*
C1—H1⋯O2^i^	0.95	2.43	3.246 (5)	144
C9—H9*B*⋯O1^ii^	0.98	2.46	3.413 (4)	163
C11—H11*C*⋯O1^iii^	0.98	2.44	3.406 (4)	168

Symmetry codes: (i) 
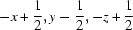
; (ii) 
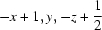
; (iii) 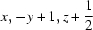.

**Fig. 1.**
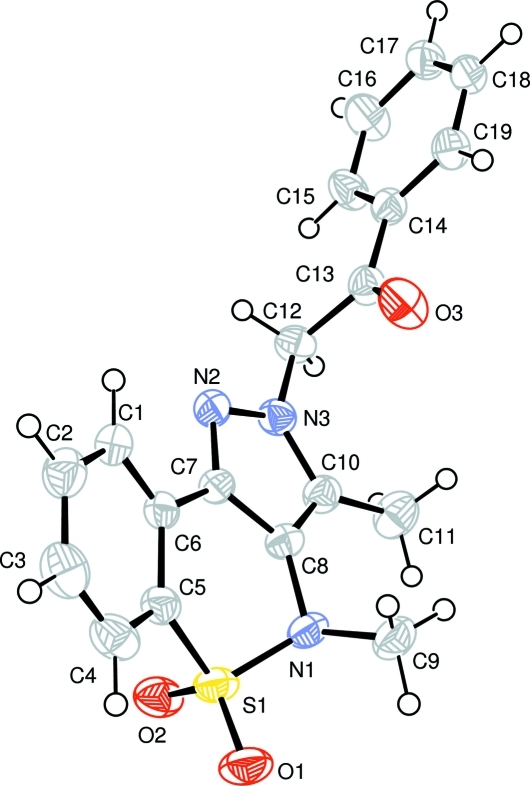
The title molecule with displacement ellipsoids plotted at the 30% probability level.

**Fig. 2.**
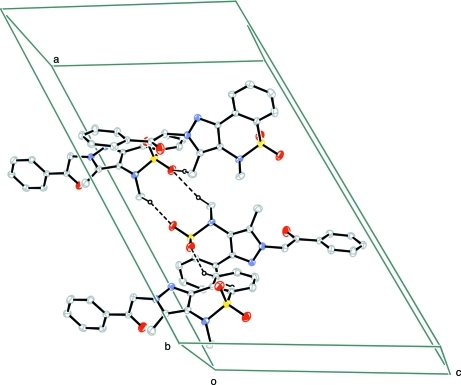
A part of the unit cell showing intermolecular hydrogen bonding interactions as dashed lines. H-atoms not involved in hydrogen bonding have been excluded for clarity.

## supplementary crystallographic information

### Comment

Both benzothiazines and pyrazoles are known as versatile biologically active heterocyclic nuclei. Pyrazoles are found to be cytotoxic agents (Ciciani *et al.*, 2008), anti-tumor (Farag *et al.*, 2008), anti-malarial (Cunico *et al.*, 2006), *etc*. In continuation of our research interests in biologically active molecules (Ahmad *et al.*, 2010), we have fused both of these heterocycles and herein report the synthesis and crystal structure of the title compound.

The bond distances and angles in the title molecule (Fig. 1) agree very well with the corresponding bond distances and angles reported in closely related compounds (Siddiqui *et al.*, 2008). The heterocyclic thiazine ring adopts a half chair conformation with atoms S1 and N1 displaced by 0.530 (5) and 0.229 (6) Å, respectively, on opposite sides from the mean plane formed by the remaining ring atoms. The ethanone group O3/C12/C13/C14 is oriented at 3.8 (3) °, with the benzene ring (C14–C19) which lies almost perpendicular to the pyrazolyl ring (N2/N3/C7/C8/C10) with a dihedral between the two planes of 89.22 (11)°. The structure is devoid of classical hydrogen bonds. However, intermolecular hydrogen bonding interactions of C—H···O type are present (Table 1).

### Experimental

Equimolar quantities of 3,4-dimethyl-2,4-dihydropyrazolo[4,3-*c*][1,2] benzothiazine 5,5-dioxide (1.0 g, 4.01 mmol) and corresponding phenacyl bromide (0.80 g, 4.01 mmol) were dissolved in acetonitrile (20 ml) followed by the addition of equimolar K_2_CO_3_ (0.55 g, 4.01 mmol). The mixture was subjected to reflux for 7 h. The completion of reaction was monitored with the help of TLC. The precipitates of the title compound were collected and washed with methanol. The crystals suitable for X-ray crystallographic analysis were grown from a solution of CHCl_3_:MeOH in 1:1 ratio.

### Refinement

Though all the H atoms could be distinguished in the difference Fourier map, the H-atoms were included at geometrically idealized positions and refined in riding-model approximation with the following constraints: C—H = 0.95, 0.98 and 0.99 Å, for aryl, methyl and methylene H-atoms, respectively. The *U*_iso_(H) were included at 1.5*U*_eq_(C methyl) or 1.2*U*_eq_(C non-methyl). The final difference map was essentially featureless.

### Crystal data

**Table:**

C_19_H_17_N_3_O_3_S	*F*(000) = 1536
*M**_r_* = 367.42	*D*_x_ = 1.394 Mg m^−^^3^
Monoclinic, *C*2/*c*	Mo *K*α radiation, λ = 0.71073 Å
Hall symbol: -C 2yc	Cell parameters from 6235 reflections
*a* = 24.380 (6) Å	θ = 1.0–27.5°
*b* = 11.141 (4) Å	µ = 0.21 mm^−^^1^
*c* = 14.996 (5) Å	*T* = 200 K
β = 120.76 (2)°	Block, colorless
*V* = 3500.1 (19) Å^3^	0.12 × 0.10 × 0.08 mm
*Z* = 8	

### Data collection

**Table:**

Nonius KappaCCD diffractometer	3970 independent reflections
Radiation source: fine-focus sealed tube	2847 reflections with *I* > 2σ(*I*)
graphite	*R*_int_ = 0.073
ω and φ scans	θ_max_ = 27.5°, θ_min_ = 2.1°
Absorption correction: multi-scan (*SORTAV*; Blessing, 1997)	*h* = −31→30
*T*_min_ = 0.975, *T*_max_ = 0.983	*k* = −14→14
12615 measured reflections	*l* = −18→19

### Refinement

**Table:**

Refinement on *F*^2^	Primary atom site location: structure-invariant direct methods
Least-squares matrix: full	Secondary atom site location: difference Fourier map
*R*[*F*^2^ > 2σ(*F*^2^)] = 0.069	Hydrogen site location: inferred from neighbouring sites
*wR*(*F*^2^) = 0.146	H-atom parameters constrained
*S* = 1.15	*w* = 1/[σ^2^(*F*_o_^2^) + (0.025*P*)^2^ + 10.0879*P*] where *P* = (*F*_o_^2^ + 2*F*_c_^2^)/3
3970 reflections	(Δ/σ)_max_ < 0.001
237 parameters	Δρ_max_ = 0.27 e Å^−^^3^
0 restraints	Δρ_min_ = −0.44 e Å^−^^3^

### Special details

**Table:**

Geometry. All e.s.d.'s (except the e.s.d. in the dihedral angle between two l.s. planes) are estimated using the full covariance matrix. The cell e.s.d.'s are taken into account individually in the estimation of e.s.d.'s in distances, angles and torsion angles; correlations between e.s.d.'s in cell parameters are only used when they are defined by crystal symmetry. An approximate (isotropic) treatment of cell e.s.d.'s is used for estimating e.s.d.'s involving l.s. planes.
Refinement. Refinement of *F*^2^ against ALL reflections. The weighted *R*-factor *wR* and goodness of fit *S* are based on *F*^2^, conventional *R*-factors *R* are based on *F*, with *F* set to zero for negative *F*^2^. The threshold expression of *F*^2^ > σ(*F*^2^) is used only for calculating *R*-factors(gt) *etc*. and is not relevant to the choice of reflections for refinement. *R*-factors based on *F*^2^ are statistically about twice as large as those based on *F*, and *R*- factors based on ALL data will be even larger.

### Fractional atomic coordinates and isotropic or equivalent isotropic displacement parameters (Å^2^)

**Table:**

	*x*	*y*	*z*	*U*_iso_*/*U*_eq_	
S1	0.37478 (4)	0.35736 (7)	0.23368 (6)	0.0377 (2)	
O1	0.41198 (11)	0.3575 (2)	0.18465 (19)	0.0511 (6)	
O2	0.33464 (11)	0.4586 (2)	0.21844 (19)	0.0470 (6)	
O3	0.42366 (12)	0.1146 (2)	0.65556 (19)	0.0555 (7)	
N1	0.42423 (11)	0.3433 (2)	0.3603 (2)	0.0348 (6)	
N2	0.31406 (11)	0.2240 (2)	0.43305 (19)	0.0333 (6)	
N3	0.35687 (12)	0.2864 (2)	0.51876 (19)	0.0337 (6)	
C1	0.26868 (14)	0.0818 (3)	0.2346 (2)	0.0356 (7)	
H1	0.2551	0.0498	0.2788	0.043*	
C2	0.24943 (16)	0.0283 (3)	0.1398 (3)	0.0445 (8)	
H2	0.2219	−0.0394	0.1190	0.053*	
C3	0.26953 (17)	0.0715 (3)	0.0750 (3)	0.0496 (9)	
H3	0.2563	0.0329	0.0106	0.059*	
C4	0.30886 (17)	0.1711 (3)	0.1036 (3)	0.0447 (8)	
H4	0.3230	0.2010	0.0595	0.054*	
C5	0.32727 (14)	0.2264 (3)	0.1974 (2)	0.0347 (7)	
C6	0.30809 (13)	0.1828 (3)	0.2652 (2)	0.0311 (6)	
C7	0.33508 (13)	0.2388 (3)	0.3667 (2)	0.0304 (6)	
C8	0.39001 (14)	0.3110 (3)	0.4110 (2)	0.0320 (6)	
C9	0.48457 (15)	0.2765 (3)	0.3945 (3)	0.0445 (8)	
H9A	0.5120	0.2825	0.4701	0.067*	
H9B	0.5067	0.3109	0.3614	0.067*	
H9C	0.4748	0.1919	0.3747	0.067*	
C10	0.40399 (14)	0.3389 (3)	0.5091 (2)	0.0354 (7)	
C11	0.45835 (17)	0.4063 (3)	0.5944 (3)	0.0501 (9)	
H11A	0.4820	0.4479	0.5668	0.075*	
H11B	0.4869	0.3501	0.6487	0.075*	
H11C	0.4420	0.4651	0.6236	0.075*	
C12	0.35231 (15)	0.2791 (3)	0.6109 (2)	0.0365 (7)	
H12A	0.3071	0.2675	0.5907	0.044*	
H12B	0.3673	0.3555	0.6499	0.044*	
C13	0.39244 (14)	0.1753 (3)	0.6804 (2)	0.0351 (7)	
C14	0.39025 (14)	0.1515 (3)	0.7764 (2)	0.0345 (7)	
C15	0.35436 (16)	0.2220 (3)	0.8039 (2)	0.0432 (8)	
H15	0.3320	0.2899	0.7632	0.052*	
C16	0.35119 (18)	0.1929 (4)	0.8912 (3)	0.0551 (10)	
H16	0.3268	0.2414	0.9102	0.066*	
C17	0.38329 (16)	0.0941 (3)	0.9503 (3)	0.0442 (8)	
H17	0.3802	0.0735	1.0091	0.053*	
C18	0.41977 (15)	0.0256 (3)	0.9241 (2)	0.0389 (7)	
H18	0.4425	−0.0417	0.9656	0.047*	
C19	0.42368 (15)	0.0537 (3)	0.8375 (2)	0.0378 (7)	
H19	0.4492	0.0063	0.8200	0.045*	

### Atomic displacement parameters (Å^2^)

**Table:**

	*U*^11^	*U*^22^	*U*^33^	*U*^12^	*U*^13^	*U*^23^
S1	0.0417 (4)	0.0377 (4)	0.0452 (5)	0.0021 (4)	0.0305 (4)	0.0067 (4)
O1	0.0548 (15)	0.0607 (16)	0.0576 (15)	0.0012 (13)	0.0430 (13)	0.0104 (13)
O2	0.0550 (15)	0.0386 (13)	0.0570 (15)	0.0097 (11)	0.0356 (13)	0.0128 (12)
O3	0.0629 (16)	0.0654 (18)	0.0503 (15)	0.0271 (14)	0.0377 (13)	0.0125 (13)
N1	0.0318 (13)	0.0371 (15)	0.0427 (15)	−0.0028 (11)	0.0243 (12)	0.0012 (12)
N2	0.0305 (13)	0.0380 (14)	0.0324 (13)	0.0011 (11)	0.0169 (11)	0.0020 (11)
N3	0.0353 (13)	0.0373 (14)	0.0313 (13)	0.0017 (12)	0.0190 (11)	0.0019 (11)
C1	0.0288 (15)	0.0397 (18)	0.0354 (17)	0.0004 (14)	0.0144 (14)	0.0027 (14)
C2	0.0388 (18)	0.0427 (19)	0.0447 (19)	−0.0034 (15)	0.0160 (16)	−0.0040 (16)
C3	0.054 (2)	0.053 (2)	0.0355 (18)	0.0026 (18)	0.0183 (17)	−0.0076 (17)
C4	0.053 (2)	0.050 (2)	0.0370 (18)	0.0111 (17)	0.0275 (17)	0.0050 (16)
C5	0.0332 (16)	0.0378 (17)	0.0358 (16)	0.0070 (14)	0.0196 (14)	0.0080 (14)
C6	0.0265 (14)	0.0351 (16)	0.0311 (16)	0.0044 (12)	0.0143 (12)	0.0051 (13)
C7	0.0276 (14)	0.0348 (16)	0.0315 (15)	0.0022 (13)	0.0171 (13)	0.0034 (13)
C8	0.0322 (15)	0.0333 (16)	0.0350 (16)	−0.0010 (13)	0.0204 (13)	0.0001 (13)
C9	0.0341 (17)	0.047 (2)	0.059 (2)	0.0022 (16)	0.0292 (17)	0.0043 (18)
C10	0.0341 (16)	0.0352 (17)	0.0391 (17)	0.0011 (14)	0.0203 (14)	0.0011 (14)
C11	0.046 (2)	0.054 (2)	0.050 (2)	−0.0121 (18)	0.0240 (18)	−0.0155 (18)
C12	0.0428 (17)	0.0383 (17)	0.0343 (16)	0.0014 (15)	0.0241 (15)	−0.0009 (14)
C13	0.0324 (15)	0.0393 (17)	0.0348 (16)	0.0031 (14)	0.0179 (14)	−0.0005 (14)
C14	0.0312 (15)	0.0403 (17)	0.0320 (16)	−0.0004 (14)	0.0162 (13)	0.0009 (14)
C15	0.0458 (19)	0.050 (2)	0.0403 (18)	0.0174 (17)	0.0265 (16)	0.0124 (16)
C16	0.058 (2)	0.070 (3)	0.049 (2)	0.025 (2)	0.036 (2)	0.015 (2)
C17	0.0404 (18)	0.057 (2)	0.0380 (18)	0.0040 (17)	0.0223 (16)	0.0093 (17)
C18	0.0358 (16)	0.0367 (17)	0.0371 (17)	0.0002 (14)	0.0136 (14)	0.0045 (14)
C19	0.0362 (17)	0.0367 (18)	0.0411 (18)	0.0059 (14)	0.0202 (15)	0.0002 (14)

### Geometric parameters (Å, °)

**Table:**

S1—O1	1.430 (2)	C8—C10	1.365 (4)
S1—O2	1.433 (2)	C9—H9A	0.9800
S1—N1	1.656 (3)	C9—H9B	0.9800
S1—C5	1.766 (3)	C9—H9C	0.9800
O3—C13	1.211 (4)	C10—C11	1.490 (4)
N1—C8	1.432 (3)	C11—H11A	0.9800
N1—C9	1.486 (4)	C11—H11B	0.9800
N2—C7	1.342 (3)	C11—H11C	0.9800
N2—N3	1.361 (3)	C12—C13	1.530 (4)
N3—C10	1.362 (4)	C12—H12A	0.9900
N3—C12	1.444 (4)	C12—H12B	0.9900
C1—C2	1.383 (4)	C13—C14	1.491 (4)
C1—C6	1.396 (4)	C14—C15	1.386 (4)
C1—H1	0.9500	C14—C19	1.387 (4)
C2—C3	1.381 (5)	C15—C16	1.388 (4)
C2—H2	0.9500	C15—H15	0.9500
C3—C4	1.383 (5)	C16—C17	1.378 (5)
C3—H3	0.9500	C16—H16	0.9500
C4—C5	1.383 (4)	C17—C18	1.374 (4)
C4—H4	0.9500	C17—H17	0.9500
C5—C6	1.406 (4)	C18—C19	1.386 (4)
C6—C7	1.454 (4)	C18—H18	0.9500
C7—C8	1.404 (4)	C19—H19	0.9500
			
O1—S1—O2	118.56 (15)	N1—C9—H9C	109.5
O1—S1—N1	107.98 (14)	H9A—C9—H9C	109.5
O2—S1—N1	107.12 (14)	H9B—C9—H9C	109.5
O1—S1—C5	109.31 (15)	N3—C10—C8	104.9 (3)
O2—S1—C5	108.32 (14)	N3—C10—C11	123.6 (3)
N1—S1—C5	104.67 (14)	C8—C10—C11	131.4 (3)
C8—N1—C9	115.7 (3)	C10—C11—H11A	109.5
C8—N1—S1	110.62 (19)	C10—C11—H11B	109.5
C9—N1—S1	117.0 (2)	H11A—C11—H11B	109.5
C7—N2—N3	103.8 (2)	C10—C11—H11C	109.5
N2—N3—C10	113.6 (2)	H11A—C11—H11C	109.5
N2—N3—C12	118.5 (2)	H11B—C11—H11C	109.5
C10—N3—C12	127.5 (3)	N3—C12—C13	111.0 (2)
C2—C1—C6	120.0 (3)	N3—C12—H12A	109.4
C2—C1—H1	120.0	C13—C12—H12A	109.4
C6—C1—H1	120.0	N3—C12—H12B	109.4
C3—C2—C1	121.1 (3)	C13—C12—H12B	109.4
C3—C2—H2	119.4	H12A—C12—H12B	108.0
C1—C2—H2	119.4	O3—C13—C14	122.5 (3)
C2—C3—C4	120.0 (3)	O3—C13—C12	119.7 (3)
C2—C3—H3	120.0	C14—C13—C12	117.8 (3)
C4—C3—H3	120.0	C15—C14—C19	119.7 (3)
C3—C4—C5	119.1 (3)	C15—C14—C13	121.7 (3)
C3—C4—H4	120.4	C19—C14—C13	118.6 (3)
C5—C4—H4	120.4	C14—C15—C16	119.8 (3)
C4—C5—C6	121.7 (3)	C14—C15—H15	120.1
C4—C5—S1	120.1 (2)	C16—C15—H15	120.1
C6—C5—S1	118.1 (2)	C17—C16—C15	120.4 (3)
C1—C6—C5	117.9 (3)	C17—C16—H16	119.8
C1—C6—C7	123.9 (3)	C15—C16—H16	119.8
C5—C6—C7	118.0 (3)	C18—C17—C16	119.8 (3)
N2—C7—C8	110.7 (3)	C18—C17—H17	120.1
N2—C7—C6	125.7 (3)	C16—C17—H17	120.1
C8—C7—C6	123.5 (3)	C17—C18—C19	120.5 (3)
C10—C8—C7	107.0 (3)	C17—C18—H18	119.8
C10—C8—N1	128.5 (3)	C19—C18—H18	119.8
C7—C8—N1	124.5 (3)	C18—C19—C14	119.8 (3)
N1—C9—H9A	109.5	C18—C19—H19	120.1
N1—C9—H9B	109.5	C14—C19—H19	120.1
H9A—C9—H9B	109.5		
			
O1—S1—N1—C8	−164.8 (2)	C6—C7—C8—C10	−174.9 (3)
O2—S1—N1—C8	66.5 (2)	N2—C7—C8—N1	179.7 (3)
C5—S1—N1—C8	−48.4 (2)	C6—C7—C8—N1	3.3 (5)
O1—S1—N1—C9	−29.4 (3)	C9—N1—C8—C10	74.8 (4)
O2—S1—N1—C9	−158.1 (2)	S1—N1—C8—C10	−149.2 (3)
C5—S1—N1—C9	87.0 (2)	C9—N1—C8—C7	−103.0 (4)
C7—N2—N3—C10	−0.4 (3)	S1—N1—C8—C7	33.0 (4)
C7—N2—N3—C12	−173.6 (3)	N2—N3—C10—C8	1.3 (3)
C6—C1—C2—C3	1.3 (5)	C12—N3—C10—C8	173.8 (3)
C1—C2—C3—C4	−0.9 (5)	N2—N3—C10—C11	−176.5 (3)
C2—C3—C4—C5	−0.3 (5)	C12—N3—C10—C11	−4.0 (5)
C3—C4—C5—C6	1.3 (5)	C7—C8—C10—N3	−1.6 (3)
C3—C4—C5—S1	−177.4 (3)	N1—C8—C10—N3	−179.7 (3)
O1—S1—C5—C4	−27.1 (3)	C7—C8—C10—C11	176.0 (3)
O2—S1—C5—C4	103.5 (3)	N1—C8—C10—C11	−2.2 (6)
N1—S1—C5—C4	−142.5 (3)	N2—N3—C12—C13	90.0 (3)
O1—S1—C5—C6	154.2 (2)	C10—N3—C12—C13	−82.2 (4)
O2—S1—C5—C6	−75.3 (3)	N3—C12—C13—O3	1.9 (4)
N1—S1—C5—C6	38.8 (3)	N3—C12—C13—C14	−176.9 (3)
C2—C1—C6—C5	−0.3 (4)	O3—C13—C14—C15	179.8 (3)
C2—C1—C6—C7	−174.6 (3)	C12—C13—C14—C15	−1.4 (5)
C4—C5—C6—C1	−0.9 (4)	O3—C13—C14—C19	−2.1 (5)
S1—C5—C6—C1	177.8 (2)	C12—C13—C14—C19	176.6 (3)
C4—C5—C6—C7	173.6 (3)	C19—C14—C15—C16	−1.2 (5)
S1—C5—C6—C7	−7.7 (4)	C13—C14—C15—C16	176.8 (3)
N3—N2—C7—C8	−0.7 (3)	C14—C15—C16—C17	−0.3 (6)
N3—N2—C7—C6	175.7 (3)	C15—C16—C17—C18	1.4 (6)
C1—C6—C7—N2	−18.5 (5)	C16—C17—C18—C19	−1.1 (5)
C5—C6—C7—N2	167.3 (3)	C17—C18—C19—C14	−0.4 (5)
C1—C6—C7—C8	157.4 (3)	C15—C14—C19—C18	1.5 (5)
C5—C6—C7—C8	−16.8 (4)	C13—C14—C19—C18	−176.6 (3)
N2—C7—C8—C10	1.5 (4)		

### Hydrogen-bond geometry (Å, °)

**Table:**

*D*—H···*A*	*D*—H	H···*A*	*D*···*A*	*D*—H···*A*
C1—H1···O2^i^	0.95	2.43	3.246 (5)	144
C9—H9B···O1^ii^	0.98	2.46	3.413 (4)	163.
C11—H11C···O1^iii^	0.98	2.44	3.406 (4)	168.

Symmetry codes: (i) −*x*+1/2, *y*−1/2, −*z*+1/2; (ii) −*x*+1, *y*, −*z*+1/2; (iii) *x*, −*y*+1, *z*+1/2.

## References

[bb1] Ahmad, M., Siddiqui, H. L., Zia-ur-Rehman, M. & Parvez, M. (2010). *Eur. J. Med. Chem.* **45**, 698–704. 10.1016/j.ejmech.2009.11.016 19962218

[bb2] Blessing, R. H. (1997). *J. Appl. Cryst.* **30**, 421–426.

[bb3] Ciciani, G., Coronnello, M., Guerrini, G., Selleri, S., Cantore, M., Failli, P., Mini, E. & Costanzo, A. (2008). *Bioorg. Med. Chem.* **16**, 9409–9419. 10.1016/j.bmc.2008.09.055 18845441

[bb4] Cunico, W., Cechinel, C. A., Bonacorso, H. G., Martins, M. A. P., Zanatta, N., Souza, M. V. N., Freitas, I. O., Soaresa, R. P. & Krettli, A. U. (2006). *Bioorg. Med. Chem.* **16**, 649–653. 10.1016/j.bmcl.2005.10.033 16257205

[bb5] Farag, A. M., Mayhoub, A. S., Barakatb, S. E. & Bayomi, A. H. (2008). *Bioorg. Med. Chem. Lett.* **16**, 881–889. 10.1016/j.bmc.2007.10.015 17962022

[bb6] Farrugia, L. J. (1997). *J. Appl. Cryst.* **30**, 565.

[bb7] Hooft, R. (1998). *COLLECT* Nonius BV, Delft, The Netherlands.

[bb8] Otwinowski, Z. & Minor, W. (1997). *Methods in Enzymology*, Vol. 276, *Macromolecular Crystallography*, Part A, edited by C. W. Carter Jr & R. M. Sweet, pp. 307–326. New York: Academic Press.

[bb9] Sheldrick, G. M. (2008). *Acta Cryst.* A**64**, 112–122. 10.1107/S0108767307043930 18156677

[bb10] Siddiqui, W. A., Ahmad, S., Tariq, M. I., Siddiqui, H. L. & Parvez, M. (2008). *Acta Cryst.* C**64**, o4–o6. 10.1107/S0108270107059173 18216455

